# Long Noncoding RNAs as Emerging Regulators of Seed Development, Germination, and Senescence

**DOI:** 10.3390/ijms26178702

**Published:** 2025-09-06

**Authors:** Adrian Motor, Marta Puchta-Jasińska, Paulina Bolc, Maja Boczkowska

**Affiliations:** Plant Breeding and Acclimatization Institute-National Research Institute, 05-870 Radzików, Poland; a.motor@ihar.edu.pl (A.M.); p.bolc@ihar.edu.pl (P.B.); m.boczkowska@ihar.edu.pl (M.B.)

**Keywords:** long noncoding RNA, seed germination, regulation of gene expression

## Abstract

Long noncoding RNAs (lncRNAs) have emerged as key regulators of gene expression during seed development and physiology. This review examines the diverse roles of lncRNAs in key stages of seed development, including embryogenesis, maturation, dormancy, germination, and aging. It integrates the current understanding of the biogenesis and classification of lncRNAs, emphasizing their functional mechanisms in seeds, particularly those acting in *cis* and *trans*. These mechanisms include the scaffolding of polycomb and SWI/SNF chromatin remodeling complexes, the guidance of RNA-directed DNA methylation, the ability to function as molecular decoys, and the modulation of small RNA pathways via competitive endogenous RNA activity. This review highlights the regulatory influence of lncRNAs on abscisic acid (ABA) and gibberellin (GA) signaling pathways, as well as light-responsive circuits that control dormancy and embryonic root formation. Endosperm imprinting processes that link parental origin to seed size and storage are also discussed. Emerging evidence for epitranscriptomic modifications, such as m6A methylation, and the formation of LncRNA–RNA-binding protein condensates that maintain resting states and coordinate reserve biosynthesis are also reviewed. Advances in methodologies, including single-cell and spatial transcriptomics, nascent transcription, direct RNA sequencing, and RNA–chromatin interaction mapping, are expanding the comprehensive lncRNA landscape during seed development and germination. These advances facilitate functional annotation. Finally, possible translational research applications are explored, with a focus on developing lncRNA-based biomarkers for seed vigor and longevity.

## 1. Introduction

Seeds are fundamental to ecosystems. They enable plants to reproduce and disperse, thereby ensuring the continuity of plant populations and the overall resilience and diversity of ecosystems [1]. Agriculture and food security depend on these factors. Seeds are so important that their development processes, from embryogenesis through maturation, dormancy, and germination or senescence, have been studied extensively and described at numerous levels. Several tightly regulated physiological and molecular processes have been identified. The functions of phytohormones and metabolic pathways have been recognized. Gene expression at each stage of seed development has also been described. However, new players emerge in this well-characterized landscape, and their roles must be revealed. One of these new elements is noncoding RNAs (lncRNAs). In recent decades, tremendous progress has been made in high-throughput nucleic acid sequencing technology. NGS technology has enabled time- and cost-effective genome characterization. Most eukaryotic genomes contain sequences that do not encode proteins; in fact, protein-coding sequences constitute only a small fraction of genomes [2]. The development of robust bioinformatics tools and advances in high-throughput sequencing technology have revealed the complexity of dynamic gene expression in plant cells and the mechanisms controlling it [3]. Extensive genomic sequencing data show that many DNA sequences in eukaryotic genomes are transcribed into RNA; less than 2% of these sequences correspond to protein-coding genes. This discovery has transformed our understanding of gene regulation, as a significant portion of eukaryotic genomes are transcribed into noncoding RNAs. Noncoding RNAs constitute more than 90% of the remaining genomic sequences and are not involved in protein coding. This group includes transfer RNA (tRNA), ribosomal RNA (rRNA), long noncoding RNA (lncRNA), small RNA (sRNA), small nuclear RNA (snRNA), and circular RNA (circRNA) [4,5,6]. Among other noncoding RNAs, lncRNAs have emerged as key regulatory molecules in plants.

This review summarizes the regulatory mechanisms of lncRNAs at different molecular levels and their regulatory role in seed development. Although the first discovery of lncRNAs in mice dates back to the 1980s and in plants to the 1990s, this RNA fraction remains relatively poorly characterized [7,8]. The regulatory network dependent on lncRNAs and their effects on seed development, germination, and senescence are not fully understood. However, further research is needed to understand how lncRNAs direct the molecular regulation of seed development. In-depth research is crucial for improving seed yield, quality, and viability for agricultural success.

## 2. Long Noncoding RNAs

Long noncoding RNAs (lncRNAs) are defined as >200-nt transcripts that lack protein-coding potential [9]. Unlike messenger RNAs (mRNAs), they contain no substantial open reading frames and exhibit heterogeneous biogenesis and structure, which complicates annotation and functional inference [10,11].

Previously, scientists thought that lncRNAs were random molecules that did not do much. However, they play crucial roles in regulating the expression of genes. Their interactions with DNA, RNA, and proteins shape chromatin architecture and modulate transcription in *cis* and *trans*. LncRNAs also influence RNA splicing, stability, and translation, contributing to nuclear condensation and organelle dynamics [12,13]. In plants, lncRNAs originate from intergenic, intronic, antisense, enhancer-proximal, and promoter-proximal regions, reflecting their diverse origins and regulation [4,14].

High-throughput sequencing and improved computational pipelines have expanded plant lncRNA catalogs to over one million entries spanning over eighty species [15]. However, functional characterization remains challenging because many lncRNAs are weakly expressed and display limited interspecies sequence conservation, although conservation can be substantial within species or subspecies [16,17]. Despite these challenges, increasing evidence implicates plant lncRNAs in gene silencing, reproductive development, flowering time control, stress responses, and other core pathways [18]. By regulating the expression of lncRNAs, researchers and breeders can increase plant resilience to environmental stresses such as drought and salinity, increase plant productivity, and improve plant adaptability.

## 3. LncRNA Biogenesis

In plants, most long noncoding RNAs are transcribed by polymerase II (Pol II) and undergo processes identical to those of mRNAs, such as attaching a 5′ cap, alternative splicing, and 3′ polyadenylation [19]. Compared with RNA Pol II, RNA polymerase III (Pol III) plays a distinct, albeit less common, role in the biogenesis of plant lncRNAs. Recent studies have shown that polymerase III (Pol III) also transcribes lncRNAs, typically resulting in short, stable, and abundant RNA molecules [20]. These Pol III-derived lncRNAs are polyadenylated less frequently than their Pol II-generated counterparts. Their expression profile suggests specialized roles, particularly in stress responses and immune regulation [20,21,22]. Two additional RNA polymerases, Pol IV and Pol V, are unique to plants. These polymerases transcribe specific lncRNAs that play a key role in RNA-driven DNA methylation (RdDM), an essential pathway for gene silencing and genome stabilization [14,23]. Pol IV and Pol V transcripts are typically poorly characterized, frequently lack polyadenylation, exhibit minimal expression, and are prone to instability [23,24]. LncRNAs transcribed by Pol IV are processed into small interfering RNAs (siRNAs). In contrast, lncRNAs transcribed by Pol V are recognized by the siRNA-Argonaute (AGO) complex, which guides the complex to target sites in chromatin and initiates gene silencing [23,25]. Moreover, lncRNAs transcribed by Pol V help modify local chromatin loops and are tissue specific. Pol V transcribes low-abundance, nonpolyadenylated scaffold RNAs. These RNAs usually have triphosphorylated 5′ ends and, less frequently, cap. Approximately three-quarters of these transcripts originate from transposable elements or other repeats, whereas the remainder originate from gene-adjacent intergenic regions. Genuine exonic or intronic Pol V transcription is uncommon [25,26]. The formation of plant lncRNAs is a complex process involving multiple RNA polymerases and posttranscriptional processes. These factors work together to ensure the diverse regulatory functions of these molecules in development and environmental responses.

## 4. LncRNA Classification

**Figure 1:**
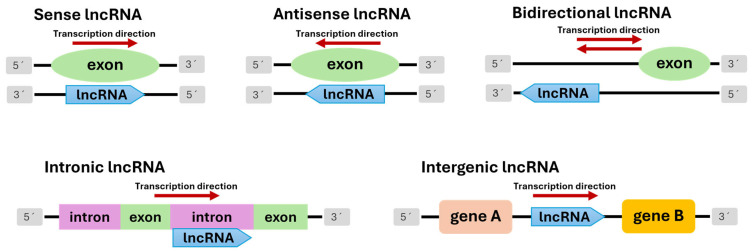
Classes of long noncoding RNAs are based on their location in the genome and orientation of transcription relative to protein-coding genes (sense lncRNAs; antisense lncRNAs; bidirectional lncRNAs; intronic lncRNAs; intergenic lncRNAs). The red arrow indicates the direction of lncRNA formation; The red arrow indicates the direction of transcription [28,29].

Elucidation of the molecular functions of plant long noncoding RNAs hinges on their classification, revealing their origin and regulatory role. It is usually based on the genomic context of plant lncRNAs with respect to protein-coding genes. For example, they may be intergenic transcripts, introns, or natural antisense transcripts. They may also be associated with regulatory elements, such as enhancers and promoters [10,20]. Recent studies indicate that the diversity of plant lncRNAs may be underestimated for two main reasons. First, there are challenges in genome annotation. Second, the loci of these molecules dynamically evolve. This underscores the importance of taking a systematic approach to identifying and categorizing them [27]. On the basis of their location in the genome and orientation of transcription relative to protein-coding genes, lncRNAs can be divided into distinct classes [23]. According to these criteria, lncRNAs can be divided into five classes (Figure 1).

### 4.1. Natural Antisense Transcripts (NATs)

NATs constitute a significant class of lncRNAs in plants. They are transcribed from the DNA strand opposite to protein-coding genes. Their sequences complement the corresponding sense transcripts and can partially or fully overlap with these protein-coding RNAs. NATs have been shown to play diverse regulatory roles, including transcriptional interference by competing with RNA polymerase II or transcription factors, masking RNA to affect mRNA stability and translation, and modulating epigenetics by recruiting chromatin-modifying complexes [27,30,31]. Approximately 70% of NATs overlap extensively with their corresponding sense transcripts in various configurations. These include tail-to-tail (3′ overlap), head-to-head (5′ overlap), and full overlap patterns. This extensive overlap can span short regions or the entire transcript length [31,32,33]. Specifically, studies have shown that most NAT pairs in *Arabidopsis* overlap in the tail-to-tail (3′ UTR) configuration. However, all other overlap types also exist, and overlapping regions often include exon sequences [31,32]. The functional interplay between NATs and their cognate genes usually results in positive or negative regulation of gene expression at the transcriptional and posttranscriptional levels. In plants such as *Arabidopsis*, maize, and rice, NATs constitute a large part of the lncRNA repertoire and control gene expression during development and in response to stress [27,34,35]. Approximately 20% of all long noncoding RNAs in the genome are of the NAT type, which regulates gene expression. One example of this is the COOLAIR in *Arabidopsis thaliana*. This COOLAIR gene regulates the *flowering locus C* (*FLC*) gene during vernalization. It plays a key role in controlling flowering time [36].

### 4.2. Sense Long Noncoding RNAs

Sense lncRNAs are transcribed from the same DNA strand as the associated protein-coding gene [12]. They often contain exons from these genes and are therefore considered to partially or entirely overlap with the protein-coding sequences of the genes from which they originate [37,38]. Sense lncRNAs may partially or entirely overlap the protein-coding sequences of the genes from which they originate. They commonly exhibit mRNA-like features, such as 5′ capping and 3′ polyadenylation, and can be multiexonic [10]. Sense lncRNAs can functionally regulate gene expression through various mechanisms, including transcriptional and posttranscriptional regulation [39]. They can influence their associated sense genes by modulating chromatin states, serving as molecular scaffolds, or affecting RNA stability and translation. Some sense lncRNAs act in *cis* to regulate neighboring or overlapping genes by promoting or repressing transcription or influencing RNA processing events [13,40].

### 4.3. Bidirectional Long Noncoding RNAs

Bidirectional lncRNAs are formed by bidirectional transcription, an inherent feature of the eukaryotic transcriptional apparatus. This phenomenon is common and conserved among eukaryotes [7]. Bidirectional lncRNAs originate near protein-coding genes’ transcription start sites (TSSs) but are transcribed from the opposite, complementary DNA strand. Thus, they run in the opposite direction of the sense (coding) transcripts adjacent to the same genomic region. These genes are characterized by either not overlapping the 5′ region of paired protein-coding genes (PCGs) or only partially overlapping it [7,19]. Unlike in animals and yeast, where bidirectional or divergent transcription at promoters is standard and well characterized, bidirectional lncRNA transcription remains unknown in plants. However, recent studies using nascent RNA sequencing techniques in *Arabidopsis* seedlings have shown that bidirectional noncoding promoters are widespread throughout the genome [41].

### 4.4. Intergenic Long Noncoding RNAs (lincRNAs)

Intergenic long noncoding RNAs (lincRNAs) are a distinct class of lncRNAs. They are defined as transcripts longer than 200 nucleotides originating from genomic regions between annotated protein-coding genes without overlapping their exons or introns. LincRNA promoters bear canonical activating histone marks (H3K4me3 and H3K36me3) but at a lower density than do mRNA promoters. This results in lower transcript abundance and extreme tissue specificity. Single-cell RNA sequencing resolved approximately 1900 lincRNAs whose expression is specific to particular developmental stages within *Arabidopsis* leaves [42]. Despite undergoing canonical capping, splicing, and 3′-end processing, weak splice site consensus sequences impede cotranscriptional splicing, contributing to half-lives that are modestly shorter than those of mRNAs [43]. Functionally, plant intergenic lncRNAs play diverse regulatory roles at the transcriptional, posttranscriptional, and epigenetic levels, and these roles are critical for modulating plant growth, development, and stress responses. LncRNAs act as molecular scaffolds, combining chromatin-modifying complexes to target loci for transcriptional regulation. They also serve as decoys or sponges, modulating the activity of microRNAs or RNA-binding proteins. Additionally, they can function as precursors for small RNAs [44]. lincRNAs account for approximately one-half to two-thirds of all plant lncRNAs, reaching ~80% in some model species [45]. While primary sequences diverge rapidly, a subset of lincRNA loci remains positionally (synthetically) conserved and retains characteristic secondary-structure motifs. This implies that selection acts on architecture rather than sequences [27]. These findings suggest that their functional importance extends beyond their primary sequence. The ability of these plants to respond to environmental stimuli, such as light, drought, and nutrient availability, demonstrates their significant role in plant adaptation [27].

### 4.5. Intronic lncRNAs (incRNAs)

IncRNAs originate entirely from intronic regions of protein-coding genes and do not overlap with exonic sequences [46]. Unlike lincRNAs, they do not cross intron–exon boundaries and are distinguished from NATs by their complete restriction to the intron space [47]. IncRNAs are generally stable. However, their half-lives are slightly shorter than those of protein-coding mRNAs. Features such as polyadenylation signals, GC content, and transcript structure influence stability [48,49]. These lncRNAs influence transcription and splicing processes and often function as regulatory molecules within the gene body, modulating the expression of their host genes. The splicing of intronic lncRNAs is crucial for their maturation and function. Although the splicing efficiency of lncRNAs can be lower than that of protein-coding genes, splicing, including the ability to influence the transcriptional activity of neighboring or host genes, is essential for their regulatory capacity [50,51]. The presence of specific splice sites and intronic splicing enhancers, for example, can regulate intronic lncRNA processing and thereby affect gene expression [52]. Additionally, intronic lncRNAs can regulate alternative splicing by interacting with splicing factors or competing for components of the splicing machinery, thereby modulating the splicing patterns of their host transcripts or other genes [53]. This multilayered regulation demonstrates how intronic lncRNAs participate in fine-tuning gene expression at the transcriptional and posttranscriptional levels.

## 5. Mechanism of Action of Long Noncoding RNAs

**Figure 2:**
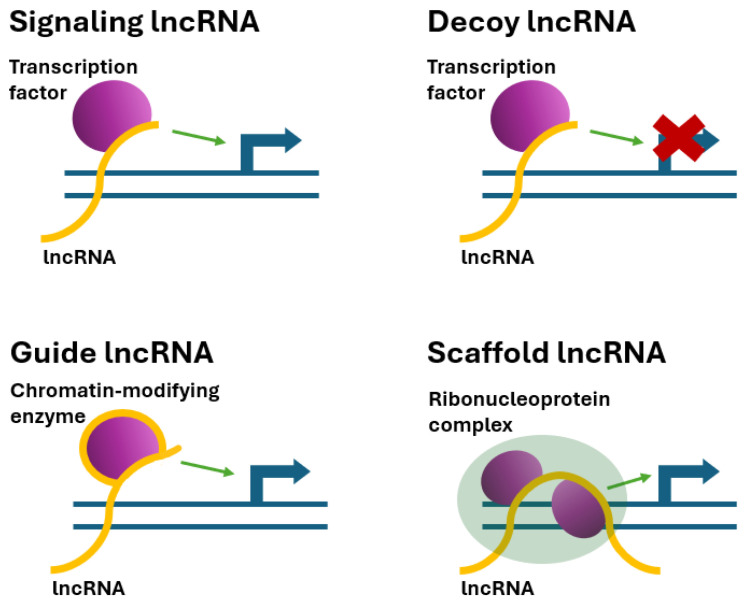
Classes of long noncoding RNAs (lncRNAs) on the basis of their mechanisms of action (signaling lncRNA; decoy lncRNA; guide lncRNA; scaffold lncRNA).

Understanding the mechanisms of action of lncRNAs is a key intermediate step between purely bioinformatic classification and functional annotation of this diverse population of transcripts. In plants, despite rapid sequence evolution, lncRNAs exhibit highly conserved rules of interaction with macromolecules that can be grouped into a limited number of molecular archetypes. A mechanistic approach, linking the structural type (lincRNA, NAT, incRNA) to the mode of action, thus allows for a better classification transfer to a functional basis. Recent reviews of the noncoding transcriptome emphasize that, in plants, lncRNAs regulate gene expression, chromatin architecture, and signaling networks at all levels of the regulatory hierarchy, from the epigenome to translation (Figure 2) [54,55].

### 5.1. Signaling lncRNAs

Signal lncRNAs act as RNA-encoded rheostats, whose abundance, isoform diversity, and chemical modifications quantitatively respond to developmental and environmental stimuli [56,57]. Their regulatory function is intrinsic to the transcript, enabling rapid induction without an additional cellular cost. A single promoter can produce multiple splicing or processing isoforms that target distinct downstream pathways [56]. Genome-wide analyses revealed that over 40% of lincRNA promoters that respond to stressors such as cold, drought, or heat are enriched with CRT/DRE, ABRE, and HSE motifs, respectively, and are characterized by H3K4me3 and H3K27ac chromatin modifications [58,59,60,61,62]. These promoters reduce nucleosome barriers at transcription start sites, facilitating the rapid recruitment of RNA polymerase II after stimulation [63]. Posttranscriptional mechanisms, including alternative splicing, polyadenylation isoforms, and reversible N^6^-methyladenosine modifications, modulate transcript stability and nuclear export [64,65]. Most plant signal-lncRNAs are under 3 kb in length, a conserved compact size that supports rapid termination and transcript clearance with minimal genomic interference [66,67]. Feedback regulation within pathways such as those controlling flowering time, abscisic acid (ABA), and reactive oxygen species (ROS) reinforces promoter activity. This is exemplified by the COOLAIR/COLDAIR–FLC and ABA-responsive DRIR loops [68]. These loops enable precise, energy-efficient environmental sensing and integration into plant gene regulatory networks.

### 5.2. Decoy (Sponge) lncRNAs

Decoy lncRNAs function as high-affinity molecular sponges that sequester regulatory factors, primarily miRNAs and RNA-binding or chromatin proteins. This renders the regulatory factors catalytically inactive. In plants, these lncRNAs block miRNA slicing via ARGONAUTE through a three-nucleotide bulge in the lncRNA–miRNA duplex and titrate proteins via short, low-complexity motifs. This occupancy prevents cleavage, translational repression, and DNA binding, thus alleviating repression across regulons. Their minimal interaction modules enable rapid evolution and diversification [9]. Stress conditions trigger transient waves of decoy lncRNA transcription from promoters marked by the activating histone modifications H3K4me3 and H3K27ac, which restrict activity to appropriate stimuli. For example, decoy lncRNAs sequester miR399 during phosphate signaling, which derepresses *PHO2* and maintains phosphate transport. Similarly, they modulate sulfate, nitrate, and potassium homeostasis [69,70]. During pathogen attack, inducible decoys redirect the transcriptional machinery to defense genes, such as *PR1*, thereby enhancing immunity [22,71]. Many decoys form phase-separated condensates that concentrate effectors and create feedback loops that stabilize responses. Consequently, decoy lncRNAs offer plants a rapid and energy-efficient way to regulate nutrient uptake, development, and immunity without incurring the costs associated with protein evolution.

### 5.3. Guide lncRNAs

Guide lncRNAs direct chromatin-modifying and transcriptional complexes to specific genomic sites, acting as RNA “GPS coordinates” in either *cis* or trans. During the vernalization process, the *cis*-acting lncRNAs COLDAIR and COOLAIR work together: COLDAIR recruits polycomb repressive complex 2 (PRC2), which deposits repressive H3K27me3 marks across the *FLOWERING LOCUS C* (*FLC*) gene. Moreover, COOLAIR promotes H3K36 demethylation and RNA polymerase II pausing, converting cold exposure into stable gene silencing [61,68,72]. The trans-acting lncRNA APOLO targets distant GA-box motifs and forms R-loops that recruit PRC1/2 to auxin- and light-responsive genes. This phenomenon modulates chromatin looping and transcription amplitude [73,74,75]. RNA polymerase V-generated lncRNAs form RNA–RNA duplexes with siRNAs and recruit the DNA methyltransferase DRM2, inducing CHH methylation of transposons and stress promoters [76,77,78]. These mechanisms, including R-loops, RNA duplexes, and multivalent protein interactions, enable lncRNAs to reprogram gene expression, encode stress memory, and mediate transgenerational epigenetic inheritance in plants via RNA-directed DNA methylation. These findings underscore the integral role of lncRNAs in dynamic chromatin routing and environmental adaptation.

### 5.4. Scaffold lncRNAs

Scaffold lncRNAs serve as organizational frameworks that simultaneously assemble multiple proteins and nucleic acids, often nucleating liquid–liquid phase-separated condensates that concentrate chromatin modifiers or signaling enzymes at specific nuclear or cytosolic sites [79,80]. Their modular RNA architecture comprises folded stem loops and intrinsically disordered, low-complexity regions that provide multiple concurrent docking sites. This allows the recruitment of histone acetyl-/methyltransferases, mediator subunits, and transcription factors within a single transcript. For example, the scaffold lncRNA LAIR’s stem–loop motifs bind to OsMOF and OsWDR5 in rice. Mutations that disrupt these motifs impair protein interactions without altering RNA levels, which separates the phenotypic effects from transcription [81]. Similarly, the pathogen-inducible ALEX1 forms phase-separated compartments with AUXIN RESPONSE FACTOR 3. Mutations that disrupt condensate formation abolish jasmonate-mediated defense enhancement despite unchanged RNA abundance [82,83]. Furthermore, RNA Pol V-derived scaffolds in *Arabidopsis* stabilize AGO4–IDN2–SWI/SNF complexes. Loss of Pol V transcription reduces SWI/SNF binding and CHH methylation, highlighting the critical structural role of scaffold lncRNAs beyond transcript quantity [84].

## 6. LncRNA in Seeds

Seed development involves embryogenesis, endosperm differentiation, maturation, desiccation, and dormancy. During these processes, lncRNAs have emerged as crucial regulators that complement—and occasionally surpass—protein-centric control (Table 1) [1].

### 6.1. Role of lncRNAs in Seed Development

#### 6.1.1. Imprinted LncRNA Sculpt Endosperm Morphogenesis

Genomic imprinting, or parent-of-origin-restricted expression, occurs predominantly in the triploid endosperm and exerts pervasive effects on seed growth, cellularization, and, ultimately, grain morphology. Studies across angiosperms have shown that lncRNAs and protein-coding genes can be imprinted and incorporated into endosperm regulatory circuits. In maize, genome-wide allelic analyses revealed extensive imprinting of noncoding RNAs, establishing a broad foundation for parental dosage effects in cereals [100,101]. Similarly, a dicot endosperm model (sunflower) revealed 36 imprinted lncRNAs that are predominantly expressed by the mother, indicating that lncRNA imprinting is not taxonomically restricted and may be a common feature of seed development [85]. A good example is the rice RNA called MISSEN (XLOC_057324). MISSEN is expressed only in the maternal allele in the endosperm, and its dosage is tightly regulated. These data show that imprinted lncRNAs regulate how the endosperm forms during development. The amount of RNA present influences this role. While some imprinted lncRNAs are positioned to act in *cis* on adjacent loci, current causal evidence in cereals highlights diverse modes—including *trans* protein sequestration—by which parent-specific lncRNAs shape the pace of nuclear division, the onset of cellularization, and ultimately, seed size and shape. We must perform systematic functional tests on the growing catalogs of imprinted lncRNAs reported in maize and sunflower endosperm to establish general principles.

#### 6.1.2. Cell Type-Specific lncRNA Networks Drive Endosperm Differentiation

Spatiotemporal and spatial transcriptomic analyses revealed that maize endosperm differentiation generates distinct anatomical and molecular compartments, including the aleurone layer (AL), the basal endosperm transfer layer (BETL), the starchy endosperm (SE), the embryo-surrounding region (ESR), and the endosperm adjacent to the scutellum (EAS). These compartments spatially segregate functions crucial for seed filling, such as nutrient transfer, storage, and protection. Single-cell RNA sequencing (scRNA-seq) reveals how these distinct cell states and their associated regulatory networks emerge during early differentiation. Spatial transcriptomics of kernels at the mid-filling stage resolves nutrient flux pathways and the spatial deployment of transporters at maternal–filial interfaces [102,103,104]. Within this structural framework, lncRNAs form a compartment-specific regulatory layer. A comprehensive survey of the AL, BETL, and SE regions identified over 1500 novel lncRNAs, approximately 82% of which exhibited spatiotemporal regulation and pronounced dynamics, particularly in the BETL region approximately 12 days after pollination (DAP) [105]. Self-organizing maps of coexpression profiles position these lncRNAs alongside protein-coding gene modules corresponding to compartment-specific functions: solute transport and cell wall biosynthesis in the BETL; defense and lipid metabolism in the AL; and storage coupled with translational machinery in the SE. These findings suggest that lncRNAs contribute to coordinating positional identity with metabolic programming. These transcriptional modules notably correlate with chromatin regions marked by H3K27me3 and imprinted loci, supporting models in which lncRNAs act both in *cis* and *trans* to modulate chromatin states during endosperm patterning [105]. Interface-specific profiling further refines this perspective. EAS, a transient subdomain of the endosperm, is enriched for the SWEET and UMAMIT transporter families. EAS also exhibits embryo-dependent transcriptional responses. This finding underscores the importance of positional cues in regulating nutrient allocation programs that may be modulated by lncRNAs [106]. Comparative analyses of sunflower endosperm support the universality of these findings, showing that lncRNAs exhibit characteristics of tissue specificity, such as short transcript length, few exons, and coexpression with genes involved in storage metabolism [85]. These conserved features highlight the widespread role of lncRNAs in synchronizing cellular differentiation with nutrient distribution. These results demonstrate that the specific lncRNA repertoires found in certain compartments are crucial for forming the maize endosperm and allocating resources. Future studies should focus on spatially restricted functional perturbations combined with RNA–chromatin interaction profiling in defined endosperm compartments to elucidate causality further.

#### 6.1.3. Testa lncRNAs Link Differentiation to Protective Metabolism

The maternal seed coats of cereal crops undergo differentiation specific to distinct cell layers and accumulate flavonoids, which contribute to pigmentation and protective functions. Recent transcriptomic atlases focused on lncRNAs in the seed coat revealed that this tissue is transcriptionally enriched with noncoding RNAs whose expression profiles closely correlate with pigment biosynthesis pathways. In Tibetan hulless barley, in-depth RNA sequencing has been conducted across developmental stages, and 9414 lncRNA isoforms originating from 6243 loci within the seed coat have been identified from color variants [107]. Among these, 1795 lncRNAs were differentially expressed between the purple and white phenotypes. The most significant transcriptional shifts occurred during the late milk and soft-dough stages, which coincided with peak anthocyanin accumulation. Genomic proximity and coexpression pattern analyses prioritized lncRNAs associated with flavonoid and anthocyanin biosynthetic genes and relevant transcription factors [107]. The current body of evidence supports a model in which testa lncRNAs form a compartment-biased regulatory stratum that interfaces with the canonical MBW transcriptional network. This interaction likely coordinates cell differentiation with specialized metabolic processes, including the biosynthesis of anthocyanins and proanthocyanidins and their intracellular transport and vacuolar sequestration [108]. Although the specific functions of individual cereal testa lncRNAs have yet to be determined, the barley lncRNA compendium provides promising candidates with sharply defined temporal expression and color-specific patterns [109]. These candidates can undergo targeted functional analysis via CRISPR interference/activation (CRISPRi/a), RNA–chromatin interaction capture (e.g., ChIRP or CHART), and spatial mapping approaches (e.g., smRNA–FISH). Through such studies, we can determine whether these lncRNAs act primarily in *cis* at pigment biosynthetic loci, serve as trans-acting modulators of transcriptional modules, or function as scaffolds that recruit chromatin-modifying complexes to stabilize coat metabolic states throughout grain filling [110].

#### 6.1.4. lncRNA Regulators Fine-Tune Lipid Deposition During Maturation

LncRNAs have been directly linked to the modulation of oil accumulation during seed maturation by interacting with transcriptional networks centered on the WRINKLED1 (WRI1) transcription factor. A comprehensive transcriptomic survey of developing seeds of *Brassica napus* identified over 8000 lncRNAs, many of which were organized into coexpression modules enriched for lipid biosynthesis pathways. Notably, these modules included lncRNAs associated with genes that encode proteins related to oil bodies and enzymes involved in triacylglycerol (TAG) metabolism, such as OLE1. This finding indicates a functional link to storage lipid networks [111]. The results of functional validation experiments further support these associations. The overexpression of the RNA MSTRG.22563 in seeds caused a 3–4% decrease in total oil content. This decrease occurred along with lower levels of WRI1 and other enzymes involved in storing fatty acids. These alterations were accompanied by metabolic shifts in glycolysis and the tricarboxylic acid (TCA) cycle, indicating a systemic reallocation of carbon resources [112]. On the other hand, overexpressing the lncRNA MSTRG.86004 increased the seed oil content by approximately 2%, although it did not affect WRI1 transcript levels. This was accompanied by delayed seed maturation, characterized by greener seeds with a higher water content and increased expression of the developmental regulator *LEC1*. These results imply that various lncRNAs can impact lipid deposition via different pathways, including regulating developmental timing [112].

Cross-species analyses reinforce the role of lncRNAs in regulating lipid metabolism. In the tung tree (*Vernicia fordii*), integrating Hi-C chromatin interaction data and transcriptomic profiles revealed numerous lncRNAs expressed in seeds that contact fatty acid and TAG biosynthetic genes. This highlights potential candidates for *cis*-acting scaffolding or distal regulatory interactions [113]. Similarly, developmental studies in tree peonies have revealed that seed-stage lncRNAs are correlated with fatty acid synthesis and TAG accumulation dynamics [114]. While direct physical interactions between plant lncRNAs and WRI1 have yet to be demonstrated, WRI1’s regulatory function in glycolysis and fatty acid biosynthesis is closely linked to the chromatin state and cofactors [91]. These studies indicate that seed-expressed lncRNAs contribute to oil accumulation by regulating WRI1 expression and the expression of its downstream lipid metabolic enzymes. These lncRNAs have predicted *cis*- or trans-regulatory relationships with fatty acid- and TAG-related genes during seed development and coordinate maturation timing with carbon partitioning and metabolic allocation. To clarify the precise causal roles and hierarchical relationships of lncRNAs within the LAFL-WRI1 regulatory network, future research employing locus-specific RNA–chromatin interaction assays combined with targeted genetic perturbations will be essential.

### 6.2. Role of lncRNAs in Seed Dormancy

Seed dormancy is a key adaptive trait that allows seeds to delay germination until favorable ecological conditions are reached, increasing plant survival and welfare [1,86]. Recent findings highlight the essential regulatory functions of lncRNAs in regulating seed dormancy through complex molecular mechanisms [1]. Specific lncRNAs influence key hormonal pathways, such as abscisic acid signaling, and interact with chromatin-modifying complexes. This affects gene expression programs that govern dormancy depth and timing. For example, the embryo-specific lncRNA VIVIPARY modulates chromatin architecture to fine-tune dormancy release in rice, whereas antisense lncRNAs regulate dormancy-associated genes, such as *DOG1*, in *Arabidopsis* [86]. These findings demonstrate that lncRNAs are key components of the complex regulatory networks that control seed dormancy. These findings have implications for improving the stress tolerance of crops and enhancing their stress tolerance. This section reviews the current knowledge of the various functions and mechanisms of lncRNAs in regulating seed dormancy by integrating findings from molecular, genetic, and epigenetic studies.

#### 6.2.1. Cis-Antisense lncRNAs Fine-Tune Master Dormancy Genes

The *DOG1* locus in Arabidopsis, the antisense lncRNA asDOG1 recruits PRC2 to repress DOG1 transcription, thereby limiting seed dormancy. Chromatin remodeling by the SWI/SNF complex further regulates asDOG1 expression, linking transcriptional interference and epigenetic silencing. Through this system, asDOG1 acts as a molecular rheostat that calibrates dormancy depth and germination timing, providing resilience against environmental fluctuations in Arabidopsis [115,116]. Recent multiomic investigations have revealed an additional regulatory layer implicating chromatin remodeling in controlling the *DOG1* locus [117]. Combining single-nucleus ATAC-seq with RNA sequencing revealed that the BRM-containing SWI/SNF chromatin remodeling complex is associated with a nucleosome-depleted region located at the 3′ terminus of the *DOG1* locus. This interaction enhances chromatin accessibility at the core promoter region of asDOG1. Functional analyses of *brm* loss-of-function mutants revealed an approximately 60% decrease in asDOG1 transcription and an increase in *DOG1* mRNA levels [117]. These findings suggest that asDOG1 production is regulated by chromatin accessibility and transcriptional interference mechanisms that integrate environmental and developmental signals within the *DOG1* regulatory network. AsDOG1 is a model *cis*-acting lncRNA scaffold that recruits PRC2. This scaffold is poised for SWI/SNF activity and senses transcriptional read-through via alternative polyadenylation. By regulating *DOG1* expression, asDOG1 calibrates dormancy depth and germination timing. This provides a molecular rheostat that buffers seeds against erratic precipitation [118]. The mechanistic principles revealed at the *DOG1* locus are emerging in other dormancy regulators, highlighting the broader importance of antisense lncRNA–chromatin assemblies in the adaptive control of plant life cycles.

#### 6.2.2. Dormancy-Enforcing lncRNAs Integrate ABA Signaling with Chromatin Repression

Dormancy-enforcing lncRNAs are crucial links between ABA-mediated hormonal signaling and chromatin-based gene regulation. They ensure seed quiescence under drought stress. Genome-wide interaction maps in *Arabidopsis* reveal over 10,000 RNA–chromatin contacts. lncRNAs are enriched at interchromosomal hubs, which reconfigure histone marks in response to environmental cues [88]. Three characterized lncRNAs illustrate how ABA perception is epigenetically integrated into seed dormancy. MUSHER is an lncRNA induced by low water potential and high temperature. It binds to the *DOG1* and *PIR1* loci. MUSHER recruits the cleavage and polyadenylation specificity factor (CPSF) to a proximal polyadenylation site in *DOG1*. This enhances *PIR1* mRNA levels and simultaneously activates *PIR1*. This dual regulation increases ABA sensitivity, reinforcing primary and secondary dormancy under drought conditions [89]. These preliminary findings demonstrate that a single lncRNA can coordinate *DOG1* expression with ABA signaling. AtR8, a Pol III-derived lncRNA in *Arabidopsis*, mediates interaction with the WRKY46 transcription factor. AtR8 facilitates the recruitment of WRKY46 to W-box motifs in the *AtEM6* promoter, increasing ABA-inducible late embryogenesis abundant (LEA) gene expression. AtR8 mutants with a loss of function show premature germination, whereas overexpression lines exhibit increased ABA sensitivity and deeper dormancy. This establishes AtR8 as a positive dormancy regulator operating upstream of chromatin-bound WRKY complexes [90]. TraesLNC1D26001.1 acts as a *cis*-activator of TaABI5, a core ABA transcription factor in wheat. Its overexpression delays radicle emergence and upregulates TaABI5, mimicking the effects of ABA treatment on seeds. Although TraesLNC1D26001.1’s chromatin effector is unknown, coexpression analyses associate it with SWI/SNF chromatin remodelers [87]. This finding suggests a mechanism similar to that of Pol II-transcribed antisense lncRNA scaffolds in dicots. Thus, these examples support a model in which ABA-inducible lncRNAs localize to chromatin or transcription factors at dormancy loci. There, they stabilize histone modifications or RNA processing states that maintain the transcriptional repression of germination. Targeting these lncRNAs through promoter engineering or RNA-guided epigenetic tools is a promising strategy for controlling preharvest sprouting and seed longevity amid climate change.

#### 6.2.3. Trans-Acting lncRNAs Rebalance ABA/GA Metabolism to Modulate Dormancy Release

The antagonistic balance between ABA catabolism and GA biosynthesis controls the release of seed dormancy. LncRNAs play pivotal trans-acting roles that link chromatin remodeling and the regulation of hormone metabolism. The embryo-specific lncRNA VIVIPARY in rice is markedly upregulated in cultivars susceptible to preharvest sprouting. The overexpression of VIVIPARY accelerated germination within 24 h post-imbibition and tripled PHS incidence, whereas the CRISPRi-mediated knockdown of VIVIPARY extended dormancy. This complex compacts chromatin at ABA signaling genes, such as *OsPYL/RCAR*. This phenomenon suppresses ABA perception and shifts the ABA:GA ratio toward germination [86]. In *Arabidopsis*, the 236-nt light-induced lncRNA HID1 accumulates in radicle nuclei following phytochrome B (phyB) activation during imbibition. HID1 binds the H3K4me3 methyltransferase ATXR7, which prevents ATXR7 from being recruited to the *NCED9* promoter. This process effectively suppresses the synthesis of ABA. In wheat, TraesLNC1D26001.1 is a dormancy-enhancing lncRNA identified via strand-specific RNA-seq. Constitutive overexpression of TraesLNC1D26001.1 elevates the ABA-responsive transcription factor *TaABI5*, delaying germination under low ABA. In contrast, the RNAi lines germinated rapidly. Coexpression and chromatin accessibility analyses associate this lncRNA with an SWI/SNF-rich module, suggesting that it modulates chromatin remodeling to amplify *TaABI5* transcription and ABA signaling [87]. These findings indicate that lncRNAs are promising targets for precision breeding. For example, engineering VIVIPARY promoters could reduce PHS in humid climates, and activating TraesLNC1D26001.1 could increase dormancy for grain storage. Future work employing single-molecule imaging and structural mutagenesis is crucial for deciphering lncRNA-effector specificity and expanding the catalog of hormone-modulating lncRNAs in crops.

#### 6.2.4. LncRNAs Act as Competitive Endogenous RNAs (ceRNAs) Within the ABA Core Circuitry

LncRNAs can interact with miRNA pathways to modulate ABA signaling in seeds. However, examples of ceRNAs that have been proven to interact directly remain rare. The most evident ABA-associated case involves the wheat lncRNA WSGAR cleaved by the seed-specific miR9678. The expression of miR9678 is reduced when WSGAR is overexpressed, resulting in decreased bioactive gibberellin, delayed germination, and increased resistance to preharvest sprouting. In contrast, silencing miR9678 accelerates radicle protrusion [95]. Although this interaction is destructive rather than protective, the crosstalk between lncRNAs and miRNAs can regulate the ABA/GA hormonal balance during the embryonic stage. The RNA-seq and overexpression data revealed a positive correlation between TraesLNC1D26001.1 and *TaABI5* transcripts and the depth of ABA-induced dormancy. Bioinformatic predictions indicate that lncRNAs and *ABI5* share a high-affinity site for miRNA9678, suggesting potential ceRNA competition [87]. However, biochemical methods (e.g., AGO immunoprecipitation (AGO-IP) and dual-luciferase assays) have not yet been reported. Therefore, TraesLNC1D26001.1 should be considered a potential ceRNA, the mode of action of which remains to be demonstrated experimentally. Madhawan et al. described lnc663 outside the ABA core, which titrates miR-1128 to derepress a PDAT-like TAG biosynthesis-related gene. This alters embryo lipid composition and indirectly influences germination kinetics [119]. In summary, the latest findings support a theory in which seed-expressed lncRNAs can control hormone metabolism in two ways: by being cut (WSGAR) or by possibly working as molecular sponges (TraesLNC1D26001.1, Lnc663). Rigorous tests, such as miRNA-decoy competition assays, loss-of-function lncRNA mutants, and quantitative measurements of miRNA loading onto AGO complexes, are now needed to substantiate ceRNA activity within the ABA network. This work will help determine whether lncRNA-guided miRNA sequestration can be used to breed crops with specific dormancy and preharvest sprouting profiles.

#### 6.2.5. Epitranscriptomic Marks Stabilize Dormancy-Associated lncRNAs

Epitranscriptomic modifications, particularly N^6^-methyladenosine (m^6^A), are crucial in stabilizing dormancy-associated lncRNAs in seeds. High-resolution m^6^A-SAC-seq profiling across nine *Arabidopsis* tissues, including dry seeds, revealed that approximately 14% of m^6^A sites are located on lncRNAs, with over 40% being specifically enriched in late-maturation and quiescent seeds [120]. These modified lncRNAs overlap with key dormancy loci, including *DOG1*, *ABI5*, and *CYP707A*. This finding suggests that m^6^A acts as a distinct regulatory layer, operating alongside transcriptional and chromatin-based controls. Functional validation involves mutants of the m^6^A writer complex, especially partial-loss alleles of the catalytic subunit MTA and its scaffold FIP37. These alleles reduce global m^6^A levels by 60–80%. These mutants exhibit premature germination after minimal after-ripening and a twofold increase in *DOG1* transcript abundance [121]. Furthermore, nanopore direct RNA sequencing links hypomethylation with decreased half-lives of seed-enriched lncRNAs containing multiple consensus DRACH motifs. This establishes m^6A as a determinant of RNA stability in dormancy [122]. The cytoplasmic reader ECT1 binds to m^6^A-rich dormancy lncRNAs during early imbibition, increasing their stability [122]. The protection of dormancy-associated lncRNAs from exonucleolytic degradation is achieved by modifications to m^6^A [92]. In vitro assays revealed that methylated synthetic asDOG1 and TraesLNC1D26001.1 transcripts resist XRN4 5′→3′ decay two- to threefold better than unmethylated versions do, and recombinant ECT2 further enhances this protection. RIP–LC–MS analyses revealed that ECT2 bound concurrently to lncRNA scaffolds and core ABA mRNAs in dry seed lysates [92]. This implies that methylated lncRNA-reader condensates may sequester hormone signaling transcripts within a long-lived ribonucleoprotein (RNP) pool, preserving dormancy competence. These findings support a hierarchical model. This model includes m^6A writers (MTA/FIP37) that install epitranscriptomic marks on dormancy lncRNAs during seed maturation, YTH readers (ECT2/3/4/1) that shield these RNAs from degradation, and stabilized lncRNAs that scaffold chromatin and RNA complexes to sustain repression of genes that promote germination. Targeted manipulation of lncRNA methylation—by editing writer components or site-specific m^6^A engineering—offers a novel strategy to modulate seed dormancy independently of protein-coding genes.

### 6.3. Role of lncRNAs in Seed Germination

LncRNAs have emerged as vital regulators of seed germination, orchestrating complex gene expression networks in response to developmental cues and environmental signals [41]. These RNAs modulate key hormonal pathways, particularly ABA signaling, and influence chromatin architecture to fine-tune germination timing [1]. Recent high-throughput studies revealed widespread noncoding transcription associated with enhancer activity, antisense transcripts, and dynamic chromatin accessibility during germination. These studies highlight lncRNAs as integral components of the molecular framework controlling seed-to-seedling transitions [1].

#### 6.3.1. Light-Responsive lncRNA Gating of the phyB–ABA/GA Module in Seed Germination

HID1, a 236-nucleotide lincRNA, was initially identified as a red-light regulator of photomorphogenesis [93]. It was subsequently shown to function in seed germination. In imbibed *Arabidopsis* seeds, a brief red-light pulse activates phytochrome B (phyB), which significantly accumulates HID1 within radicle nuclei over 24–48 h [2,94]. Although phyB does not directly bind HID1, light-activated phyB enhances HID1 transcription and/or RNA stability, positioning HID1 downstream in the phyB signaling cascade that controls germination [94]. Consequently, *hid1* loss-of-function mutants accumulate elevated ABA levels, whereas wild-type seeds exposed to red light rapidly decline in ABA levels alongside HID1 induction [2]. Global transcriptomic analyses revealed the coordinated regulation of hormone metabolism genes by HID1 and phyB: ABA biosynthesis and signaling components are downregulated, whereas GA biosynthetic genes, including *GA20ox* and *GA3ox*, are upregulated. This shift in the ABA:GA balance favors germination [2]. Phenotypically, *hid1* mutants exhibit delayed radicle protrusion under “phyB-on” (high red:far-red) conditions. Conversely, phyB-dependent HID1 overexpression mitigates ABA-induced germination inhibition, restoring near-normal rates [94]. HID1 is a pivotal early molecular switch that translates light perception into the repression of ABA synthesis and the promotion of GA production. This enables embryo dormancy release. The *HID1–NCED9* axis exemplifies an lncRNA-mediated intersection between environmental cues and chromatin regulation, broadening the classical phyB–PIF signaling framework to incorporate RNA-based control layers that govern seed germination.

#### 6.3.2. Auxin-Linked lncRNA Control of Seed Vigor

The endosperm-specific long noncoding RNA SVR (seed vigor-related) has been identified as a key positive regulator of seed vigor in rice. The predominant SVR transcript is 889 nucleotides long. It maps near the *SAUR* gene cluster on chromosome 9, approximately 19.6 kilobases (kb) upstream of *OsSAUR55*. SVR expression peaks in the endosperm during early imbibition, coinciding with the temporal induction of multiple SAUR paralogs [123].

Using two independent CRISPR/Cas9 SVR knockout lines (CR-SVR-8 and CR-SVR-11), functional genetics demonstrated a significant delay in germination rates under both normal (25 °C) and chilling (15 °C) conditions—a 30–40% reduction [123]. While SVR overexpression remains unexplored, the inverse correlation between SVR levels and germination speed suggests that SVR acts as a positive regulator of seed vigor. Bioinformatic network analysis revealed that the SVR is centrally located within a *cis*-regulatory module that links it to auxin signaling. Nineteen adjacent *SAUR* genes are predicted to be direct targets of SVR, and their expression is markedly downregulated in SVR knockout mutants [123]. Disruption of *OsSAUR33* in rice similarly impairs seed vigor by affecting sugar remobilization and SnRK1A signaling during germination [123]. Future studies should investigate whether SVR mediates its effects by forming RNA–DNA hybrids or recruiting chromatin-modifying complexes to the *SAUR* locus and whether auxin-responsive transcription factors, such as ARF10 or ARF16, participate in this regulatory network. These investigations elucidate how an endosperm-restricted lncRNA integrates hormonal and metabolic signals to fine-tune germination timing. This presents a promising target for enhancing seed vigor under variable field temperatures.

#### 6.3.3. Thermoresponsive lncRNA Networks Recapitulate Dormancy Under Heat

Elevated temperatures during the late grain-filling stage (35 °C during the day and 25 °C at night for 21–35 days after anthesis) significantly reduce primary dormancy in wheat, increasing susceptibility to preharvest sprouting [124]. Transcriptome analysis of the landrace Waitoubai under heat stress conditions revealed 273 differentially expressed lncRNAs (DE-lncRNAs) and changes in mRNAs, miRNAs, and circRNAs [125]. Integrative coexpression network analysis grouped these genes into 13 modules. Notably, the red and turquoise modules, which are positively correlated with heat-induced dormancy loss, were enriched with GA biosynthesis genes, such as *TaGA20ox1*, and ABA catabolism genes, such as *TaCYP707A1* and *TaCYP707A4*. Six hub lncRNAs within these modules exhibited strong *cis*- or trans-interactions with hormone pathway genes. These lncRNAs also contained predicted RNA-binding protein and miRNA interaction motifs. These findings suggest that these genes act as thermal sensors that modulate the ABA/GA balance during germination [124]. One DE-lncRNA, TahlnRNA27, which is rapidly induced by a 37 °C shock and was previously annotated as an miR2010 precursor, was found to be upregulated at 35 °C. It also colocalizes with the GA catabolic gene *TaGA2ox5*, reinforcing the connection between heat responsiveness in lncRNAs and GA metabolism [126]. These lncRNAs bias gene regulatory networks toward GA accumulation and ABA depletion via RBP scaffolding or miRNA competition, thereby alleviating seed dormancy. Forward genetic manipulations, such as CRISPR interference or antisense knockdown of the identified hub lncRNAs, are necessary to establish causal relationships and assess their agronomic utility in mitigating PHS under rising temperatures. This integrated transcriptomic and network analysis implicates lncRNAs as pivotal modulators that connect environmental temperature cues to the hormonal control of dormancy release in wheat. These lncRNAs offer promising targets for crop improvement in the context of climate change adaptation.

#### 6.3.4. Single-Cell Atlases Reveal Cell Type-Specific lncRNA Bursts

Recent single-cell and single-nucleus transcriptomic atlases have transformed our understanding of lncRNA dynamics during the seed-to-seedling transition. High-resolution 10x Genomics scRNA-seq time-course data spanning imbibition to radicle emergence revealed that embryo cells initially pass through a homogeneous “boot” transcriptional state. This state coincides with chromatin decondensation and metabolic reactivation, which are especially prominent in the nascent vasculature [99]. While mRNAs are predominant, more than 5% of transcripts during this stage originate from noncoding loci, suggesting the early and widespread presence of lncRNAs. Integrative csRNA-seq and ATAC-seq profiling revealed the presence of 899 antisense lncRNAs, 2702 antisense noncoding TSSs, and 2841 intergenic noncoding TSSs [41]. Among these peaks, approximately 45% peak within 6–26 h post-imbibition, which coincides with exit from the boot state. This results in more than 2000 pronounced “upspikes” in lncRNA expression during early germination. Motif enrichment links these lncRNA promoters to the ABI5/ABF and RAP2.1 transcription factor modules, connecting them to the rapid decrease in ABA sensitivity characteristic of germination progression [41]. Upon lineage specification, the expression of lncRNAs becomes highly tissue specific, as demonstrated by trajectory analyses. The repertoires of vascular initials, protoderms, and scutellum cells exhibit less than 10% overlap in their respective lncRNA expression patterns [42]. Gene regulatory network inference places many lncRNAs at nodes with high betweenness, which bridge modules for ribosome biogenesis, oxidative phosphorylation, and amino acid metabolism. These processes drive the translational surge underlying radicle protrusion [42]. Candidates such as lnc-RADICLEUP1 and lnc-VACUO1 physically interact with DEAD-box RNA helicases and m^6^A reader proteins, suggesting direct involvement in RNA processing and stability. Spatially resolved nascent RNA fluorescence in situ hybridization confirmed the rapid, cell type-specific accumulation of these lncRNAs: lnc-VACUO1 localizes to the protoderm, and lnc-BOOT163 localizes to vascular cells within 90 min of imbibition [42].

#### 6.3.5. Stress-Adaptive lncRNAs Modulate Reactive-Oxygen Homeostasis

ROS accumulate in dry seeds where mitochondrial activity is halted. ROS levels increase again during imbibition-associated respiratory bursts. Recent genome-wide RNA-seq analyses have revealed that lncRNAs are an underappreciated layer in seed redox regulation. For example, the artificial aging of rice seeds at 43 °C with 85% relative humidity for eight days induced the expression of 284 lncRNAs, many of which were coexpressed with genes in the antioxidant pathway, such as those encoding glutathione, peroxiredoxin, and thioredoxin components [97]. Twenty-seven lncRNAs were among the top five percent of hub nodes in the ROS detoxification network. Similarly, 128 aging-responsive lncRNAs, including antisense transcripts that overlap with peroxiredoxin and glutathione-S-transferase loci, were identified in *Metasequoia glyptostroboides*. This suggests direct *cis*-regulation of antioxidant gene expression [127]. Structural predictions suggest extensive RNA–RNA complementarity between antisense lncRNAs (e.g., anti-PRXII–AS1) and their target antioxidant mRNAs. This finding is consistent with the idea that antisense lncRNAs play a *cis*-stabilizing role against nuclease-mediated decay, which accelerates during seed aging [128]. Although there is limited experimental evidence of RNA pull-down in seeds, motif enrichment in ROS-related lncRNAs supports this model [129]. Promoter analyses showing the overrepresentation of ABA-responsive ABI5/ABF motifs in rice ROS-hub lncRNAs suggest their integration with hormonal networks. The predicted targets include *OsGPX3* and *OsPRX25*, which position lncRNAs at the interface of ABA-ROS crosstalk that regulates dormancy release [128]. In wheat, heat-dormancy datasets document the coinduction of lncRNA-*GA20ox* modules alongside decreased levels of transcripts that scavenge ROS, implying that lncRNAs can modulate hormonal and redox equilibrium to favor either dormancy or germination [124]. These findings support a theory in which early-stage lncRNAs preserve redox balance by making antioxidant mRNAs more stable in *cis* or trans, changing chromatin at locations of detoxification genes, and connecting ABA/GA signals to redox set-points. Advanced in vivo RNA structural mapping and CRISPR/dCas13-mediated perturbations at the level of a single embryo will be vital for defining these mechanisms and evaluating the potential of editing redox-associated lncRNAs to increase seed longevity or optimize seedling emergence under climate-related oxidative stress.

### 6.4. Role of lncRNAs in Seed Senescence

Long noncoding RNAs are increasingly recognized as crucial seed senescence regulators that influence gene expression and the epigenetic states determining seed longevity and viability [130]. During seed aging, these RNAs mediate chromatin remodeling, transcriptional control, and posttranscriptional mechanisms in response to developmental cues and environmental stressors. Plant studies have revealed that lncRNAs modulate key longevity-associated pathways by integrating hormonal signals and chromatin dynamics to maintain seed vigor. Elucidating the functions of lncRNAs in seed aging enhances our understanding of the molecular aging process and provides potential targets for enhancing seed storage and stress resistance in crops [98].

#### 6.4.1. Global Attrition of the lncRNA Transcriptome During Aging

Seed deterioration is driven primarily by accumulated oxidative damage, which causes DNA, RNA, and protein strand breaks, compromising cellular integrity. LncRNAs are particularly susceptible due to their low abundance, minimal RBP protection, and simpler secondary structures. This makes them prime targets for ROS-induced cleavage and exonucleolytic decay [1,28]. Strand-specific RNA-seq of artificially aged rice embryos (50% germination threshold) revealed 6002 lncRNAs, 458 of which were differentially expressed, and 99% were downregulated. This decline is correlated with reduced glutathione-disulfide reductase activity, linking lncRNA loss to compromised antioxidant defenses [97]. The functional annotation of the downregulated lncRNAs revealed that their targets included DNA excision repair enzymes, base-excision glycosylases, and thiol-redox buffering components. These findings suggest that the loss of lncRNAs exacerbates deficits in genome maintenance. A parallel PacBio and Illumina transcriptome profiling study of aged *Metasequoia glyptostroboides* seeds revealed 457 lncRNAs, 128 of which were significantly altered and predominantly downregulated. The target genes cluster in ROS detoxification pathways (e.g., peroxiredoxin and glutathione-S-transferase) and MAPK cascades that initiate PCD. A competing ceRNA network implicates lncRNA_00185 as a decoy for miRNA167, stabilizing *RCD1* transcripts that promote seed longevity [127]. Alternative splicing of the rice lncRNA LNC_037529 increases with age, generating isoforms with greater double-stranded content that may better recruit stabilizing RBPs [97]. This finding shows that keeping lncRNA molecules intact is crucial for long-term longevity of seeds. This study also reveals new ways to identify better biomarkers and improve crops.

#### 6.4.2. A Small Cohort of Longevity-Associated lncRNAs Resists Decay

A small subset of lncRNAs defies the decline observed during seed deterioration by increasing abundance and acting as molecular protectors against oxidative damage. Strand-specific RNA sequencing (RNA-seq) of artificially aged rice embryos at the 50% germination endpoint revealed only four upregulated lncRNAs (LNC_001951, Os02t0591850-01, Os03t0332600-01, and Os01t0704250-00) out of 458 differentially expressed species [97]. The target genes of these lncRNAs are enriched in pathways related to homologous recombination, mismatch repair, and mRNA surveillance. LNC_001951’s predicted *cis* targets encode DNA repair proteins, including an exonuclease and a Pol I-like DNA polymerase. These findings suggest the selective retention of repair-associated lncRNAs that mitigate the accumulation of strand breaks during aging [97]. A similar pattern was observed in *Metasequoia glyptostroboides*, where only one of the 128 age-responsive lncRNAs, termed lncRNA_00185, exhibited sustained upregulation [127]. Analysis of the ceRNA network linked lncRNA_00185 to the oxidative stress gene *RCD1* via sequestration of miR167. Degradome data revealed that miR167 binds preferentially to lncRNAs rather than *RCD1* mRNAs. VIGS of lncRNA_00185 reduced *RCD1* transcript levels by 38%, increased hydrogen peroxide by ~20%, and shortened the germination half-life under accelerated aging [127]. From an applied perspective, these “longevity lncRNAs” serve as promising early viability biomarkers because they increase as most lncRNAs and mRNAs degrade, indicating critical viability thresholds. Furthermore, CRISPR-based promoter activation of orthologs of lncRNA_00185 or m^6^A-mediated stabilization of rice repair-associated lncRNAs could strengthen intrinsic defense pathways without altering coding sequences. This offers a new way to increase seed longevity for gene banks and agriculture. This finding highlights the importance of keeping specific molecules in seeds to help them stay strong. It also allows us to develop new ways to grow plants that can better handle how their seeds are stored.

#### 6.4.3. Alternative Splicing Intensifies Under Aging Stress

Long-read and strand-specific RNA-seq analyses revealed that seed aging significantly increases the number of noncanonical splice variants of lncRNAs, adding a flexible posttranscriptional control layer to viability. In artificially aged rice embryos, 458 of the 6002 lncRNA loci presented differential expression [97]. The key longevity lncRNA LNC_037529 produced five age-specific isoforms (A1–A5) involving intron retention and exon skipping. These events modify the 5′ stem loop of LNC_037529, which is predicted to bind the DNA repair scaffold OsXRCC1. The longest isoform, 8.2 kb, accumulates exclusively in aged endosperm, suggesting enhanced structural resistance to oxidative damage [97].

PacBio sequencing detected 457 lncRNAs, 27% of which underwent isoform switching, in naturally aged seeds of dawn redwood (*Metasequoia glyptostroboides*) [131]. The longevity-associated lncRNA_00185 acquires a 72-nt retained intron that contains a high-affinity microRNA 167 (miRNA167) binding site. This strengthening of the miRNA decoy function protects RCD1 transcripts and maintains ROS scavenging. VIGS of this long isoform hastens viability loss by 18 h; the short isoform lacks this effect [131]. Aging embryos exhibit a 25–30% decrease in adenylate energy charge. Concurrently, the frequency of splice sites shifting from the canonical GU–AG to GC–AG increases in lncRNAs but not in mRNAs, implying that low-energy spliceosomes favor alternative splicing, which produces GC-rich, stem–loop-dense isoforms with improved resistance to ROS-mediated cleavage [1,97]. In silico predictions suggest that retained introns create new linear motifs for RNA-binding proteins (RBPs), including DEAD-box helicases and heterogeneous nuclear ribonucleoproteins (hnRNPs). These motifs can reshape lncRNA–protein condensates, stabilizing DNA repair and antioxidant mRNAs. Consequently, cis/trans targets of splicing-altered lncRNAs are enriched for base-excision repair (BER) and glutathione pathway genes [97]. The promoters of isoform-switching lncRNAs are enriched for ABA-responsive ABI5/ABF and heat-shock factor HSFA2 binding motifs. This suggests that hormonal and thermal signals preemptively prime splicing plasticity to buffer against oxidative stress as seed reserves diminish [1,131]. These findings show that alternative splicing is similar to an energy-sensitive control switch that adjusts the structure, interaction ability, and stability of long noncoding RNAs. This helps fine-tune plants’ antioxidant defenses and genome maintenance when the usual surveillance does not work well during seed aging. Targeting splice sites or branch-point elements in longevity-associated lncRNAs could enhance endogenous protection without disrupting protein-coding sequences.

#### 6.4.4. LncRNA-Centered ceRNA Networks Balance Antioxidant Defense and PCD

A high-resolution, full-length transcriptomic analysis of *Metasequoia glyptostroboides* seeds revealed a complex ceRNA network. This network involves lncRNAs, miRNAs, and redox-related mRNAs. It balances antioxidant defense and PCD during seed aging. The core hub consists of 18 lncRNAs, 38 miRNAs—including the dominant families miR164, miR167, and miR399—and 69 mRNAs that encode ROS scavengers, such as peroxiredoxins and glutathione-S-transferases, as well as PCD regulators, including *RCD1* and *BAG6* [127]. Each guard lncRNA contains multiple canonical binding sites for one or more hub miRNAs, which enables the sequestration of these miRNAs away from their target antioxidant or prosurvival mRNAs. Promoter motif enrichment for ABA-responsive elements (ABREs) and heat shock factors links lncRNA expression to hormonal and thermal stimuli that precede oxidative stress during storage [127,131]. Functional validation of two hub lncRNAs—Mglnc0392 (antisense to *GSTU4*) and lncRNA_00185 (an miR167 decoy that regulates *RCD1*)—by VIGS results in elevated hydrogen peroxide (~20–25%) and increased caspase-like DEVDase activity within 48 h of artificial aging [131]. Conversely, transient overexpression of lncRNA_00185 reduces ROS accumulation and delays nucleosomal DNA fragmentation. These findings demonstrate the direct modulation of PCD onset via ceRNA buffering. Network analyses identified lncRNA_00185 and Mglnc0392 as high-betweenness “circuit breakers” in the regulatory network. Comparative genomics detected selective sweeps at the promoters of miR167-decoy lncRNAs across gymnosperms and cereals, reflecting the convergent conservation of ceRNA modules that increase seed longevity. Translationally, CRISPR-mediated activation of the promoter of the rice ortholog LNC_037529 extends seed viability without affecting vegetative growth. These findings underscore the potential of harnessing lncRNA-centered ceRNA networks to improve crop and enhance seed storage resilience. This integrated lncRNA–miRNA–mRNA crosstalk constitutes a finely tuned molecular circuit that balances antioxidant defenses and programmed cell death, safeguarding seed viability during aging and under environmental stress.

#### 6.4.5. Domestication Reshaped lncRNA Loci Governing Storability

Artificial selection during the domestication of cereals and modern breeding has consistently favored rapid and uniform seed emergence. This often reduces seed dormancy and storability in exchange for faster germination. For example, the key dormancy regulator Sdr4 clearly shows signs of artificial selection in rice, reflecting breeders’ preferences to reduce dormancy and minimize preharvest sprouting [132]. In addition to protein-coding genes, lncRNAs have emerged as direct targets of domestication [133].

Furthermore, genome-wide surveys have revealed that conserved lncRNAs are linked to domestication-related traits, suggesting that noncoding loci are recurrent targets during crop improvement [134]. The extensive variation in lncRNAs across diverse rice and wheat germplasms and low sequence conservation suggest that abundant regulatory diversity is available for selection [135]. These findings support a parsimonious model in which seed performance is partly modified through the tuning of noncoding regulatory hubs, including the positioning of lncRNAs adjacent to or acting on hormones and redox-related genes critical for longevity. While direct evidence of selective sweeps at lncRNA promoters controlling oxidative stress genes is limited, the convergence of selection on dormancy, genome-wide lncRNA expression shifts, and redox control as a key factor in seed storability strongly motivates targeted genomic scans for sweep signatures and allelic diversity at longevity-associated lncRNA promoters. From a translational perspective, editing lncRNA promoter regions to modulate their dosage without altering protein-coding sequences offers a promising, pragmatic way to extend seed shelf life with minimal pleiotropic risk, especially when lncRNAs negatively regulate PHS or positively enhance ROS buffering [133,136].

## 7. Methods for Identifying Long Noncoding RNAs

**Figure 3:**
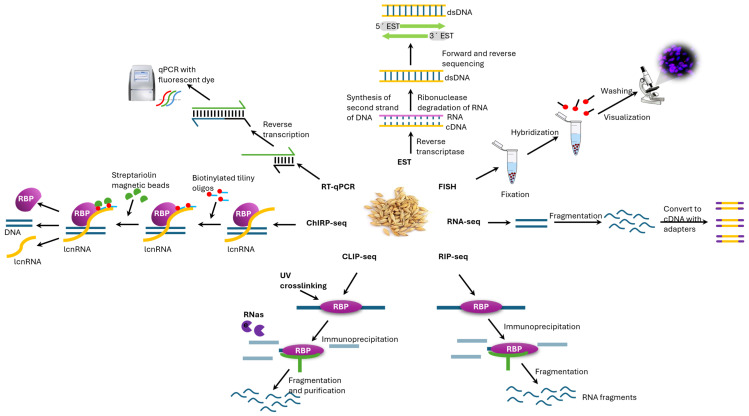
General methods for identifying long noncoding RNAs (lncRNAs) [1,140,141].

A general strategy for identifying lncRNAs involves high-throughput screening to detect their expression and determine their biological function and mechanism of action on target genes [137]. The most common methods for analyzing lncRNAs are microarray analysis and RNA sequencing. Off-the-shelf identification chips generated via microarray technology are also available for analyzing lncRNAs in *Arabidopsis*. Third-generation identification methods based on PacBio and Iso-Seq sequencing technologies and Oxford Nanopore Technologies (ONT) enable the sequencing of full-length RNA transcripts without folding. This improves the accuracy of identifying lncRNAs. RT–qPCR and fluorescence in situ hybridization can be used to verify identified lncRNAs [138]. Methods based on RNA-binding protein (RIP) immunoprecipitation, chromatin immunoprecipitation (ChIRP), and cross-linking immunoprecipitation (CLIP) can also be used to verify lncRNA targets. Fluorescence in situ hybridization (FISH) is used for localization studies [139] (Figure 3).

### 7.1. Hybridization and Sequence-Tag Approaches

Before the dominance of high-throughput RNA-seq, genome-wide tiling arrays were instrumental in revealing pervasive noncoding transcription in *Arabidopsis*. RNA from exosome-compromised plants, for example, hybridizes to tiling arrays and reveals numerous unannotated and antisense transcripts, providing an early glimpse of the plant noncoding transcriptome [142]. The At-TAX tiling array atlas expanded this mapping to 11 tissues and identified over 1000 unannotated transcribed regions [143]. A custom microarray (ATH lincRNA v1) with 60-mer probes was used to profile several thousand long intergenic noncoding RNAs (lincRNAs), including those of stress-responsive species [144]. In parallel, sequence-tag technologies provide digital expression readouts and promoter mapping. SAGE and MPSS quantified transcript abundance and detected tags from intergenic loci. Moreover, CAGE/PEAT-seq precisely delineated transcription start sites and promoter usage genome wide [145,146]. These platforms established the first systematic catalogs and promoter maps of plant lncRNAs, albeit with limited isoform resolution relative to contemporary long-read sequencing.

### 7.2. Short-Read RNA-Seq

Strand-specific Illumina RNA-seq is essential for discovering plant lncRNAs because it provides high-depth, strand-aware counts reliably quantifying low-abundance transcripts. Typical short-read datasets produce reads of ~50–150 bp, which is adequate for differential expression analysis but insufficient for isoform reconstruction and long-range splicing analysis [147]. In *Arabidopsis*, short-read compendia coupled with coding-potential filters, such as CPAT and CPC2, have substantially expanded lncRNA annotations and uncovered widespread NATs, exemplified by NATs that modulate their cognate sense genes [148,149]. Community databases, including PLncDB v2.0, which aggregates over 13,000 RNA-seq libraries across 80 species, further systematize these annotations for comparative analyses [150]. Since read fragmentation hinders isoform-aware inference, the best practice is to integrate short reads with long-read sequencing or targeted assays when the transcript structure is central to the hypothesis.

### 7.3. Long-Read and Direct RNA Sequencing

High-fidelity PacBio Iso-Seq technology provides full-length cDNA whose circular consensus accuracy improves the number of splice junction calls and isoform discrimination. This conclusion is supported by multicenter benchmarks showing that read length and accuracy primarily determine correct transcript reconstruction, whereas additional depth mainly improves quantification [151]. The Oxford Nanopore supports both cDNA and direct RNA sequencing (dRNA-seq). The latter interrogates native RNA, thereby preserving base modifications. In plants, dRNA-seq has mapped the complexity of the *Arabidopsis* transcriptome and RNA modification signatures at the single-molecule level. Similarly, in human cells, native RNA reads have enabled the discovery of isoforms and the analysis of modifications [152,153]. In practice, hybrid frameworks that integrate long reads with Illumina short-read compendia yield the most comprehensive lncRNA annotations, as exemplified by the *Arabidopsis* AtRTD3 reference, which increased differential expression, alternative splicing, and transcript boundary inference [154]. Continued development of long-read-aware algorithms (e.g., isoform callers and quantifiers) will further expand isoform-resolved lncRNA analyses in plants.

### 7.4. Expression and Localization Validation

RT–qPCR remains the standard orthogonal assay for confirming the differential expression of plant lncRNAs, as stable reference genes are normalized. Community best-practice papers formalize these procedures [155]. Spatial localization is resolved by single-molecule RNA fluorescence in situ hybridization (smFISH), which visualizes individual RNA molecules in fixed tissues. In *Arabidopsis*, smFISH detected the antisense lncRNA COOLAIR at the FLC locus and demonstrated mutual exclusivity between COOLAIR and FLC sense transcription at single alleles [72,156]. Recent whole-mount smFISH protocols extend this capability to intact organs, enabling quantitative counting and multiplexing without sectioning. HCR-RNA-FISH variants further increase sensitivity to low-abundance transcripts [157,158]. Foundational plant smFISH methods provide validated pipelines for probe design and image analysis for single-molecule quantification in *Arabidopsis* tissues [159,160]. RT–qPCR was used to validate expression claims in practice, whereas smFISH provides cellular and subcellular context. Together, they enable rigorous inference about whether an lncRNA acts locally in *cis* at chromatin or functions in *trans* from the cytoplasm [161].

### 7.5. RNA–Protein Interactome Mapping

RIP-seq remains a sensitive method for delineating plant RBP regulons, with or without formaldehyde crosslinking. In *Arabidopsis*, RIP-seq of the SR-like factor SR45 recovered over 4000 associated RNAs, revealing extensive postsplicing control of ABA pathway transcripts [162]. In contrast, UV-based CLIP methods pinpoint contact sites at near-nucleotide resolution. Early HITS-CLIP of hnRNP-like HLP1 revealed A/U-rich binding around cleavage and polyadenylation regions, linking it to pervasive alternative polyadenylation [163]. The subsequent adaptation of iCLIP to plants, and more recently, the optimized plant iCLIP2 workflow, has enabled the creation of robust, transcriptome-wide binding maps directly in plant tissues [164,165]. These tools are crucial for identifying targets of the m^6^A reader ECT2 and integrating iCLIP with other assays to determine consensus recognition and the role of intrinsically disordered regions in RNA engagement in vivo [166]. In practice, RIP-seq provides comprehensive target catalogs, including potential lncRNA partners, whereas CLIP variants furnish spatially precise interaction landscapes. Combining these methods offers coverage and mechanistic resolution for plant lncRNA–protein complexes [167].

### 7.6. RNA–Chromatin Target Mapping

ChIRP/ChIRP-seq uses pools of biotinylated antisense oligonucleotides to purify endogenous RNA–chromatin complexes, enabling genome-wide maps of RNA occupancy and copurified proteins. ChIRP-seq has defined the chromatin action of key lncRNAs in plants: APOLO engages multiple distant targets to influence 3D topology and transcriptional outputs. COLDAIR, meanwhile, is enriched across the FLC locus during vernalization, which is consistent with its role in Polycomb-mediated silencing [75,168]. Complementary RNA-centric capture methods broaden the toolkit. CHART uses hybridization capture to recover RNA-bound DNA and proteins. It was introduced to map the genomic binding of lncRNAs, such as chromatin immunoprecipitation (ChIP) for proteins. RAP uses long, tiled probes and stringent washes to map RNA–DNA contacts with high specificity. This has been demonstrated with Xist coating of the inactive X chromosome [169,170]. These assays and downstream proteomics (e.g., ChIRP-MS) provide alternative ways of localizing plant lncRNAs on chromatin and identifying their associated effectors. This allows us to determine where and how lncRNAs act in epigenomic regulation [171].

### 7.7. Key Caveats and Best Practices

Microarrays and whole-genome tiling arrays are foundational but cannot resolve isoforms or exact transcript ends. This has motivated a shift to sequencing-based catalogs [147]. Although strand-specific short-read RNA-seq provides deep and accurate quantification, the short read length limits isoform reconstruction and alternative splicing. Accordingly, long-read (PacBio/ONT) augmentation and hybrid strategies improved splice junction fidelity and transcript models [147,172]. ChIRP/ChIRP-seq is explicitly RNA-centric. It enriches endogenous RNA–chromatin complexes to map genomic occupancy and associated proteins. This process should not be confused with chromatin immunoprecipitation, which is protein-centric [171]. Notably, mechanistic assignment requires orthogonal validation. Single-molecule RNA FISH, including whole-mount protocols, localizes lncRNAs and resolves allele-specific transcription (e.g., COOLAIR at FLC). Genetic perturbations, such as CRISPRi/a or RNA-targeting screens, test necessity and sufficiency in vivo [156,173]. Adhering to this multimodal framework minimizes methodological bias and yields reproducible, high-confidence inferences about plant lncRNAs.

## 8. Bioinformatics Analysis of Long Noncoding RNAs

**Figure 4:**
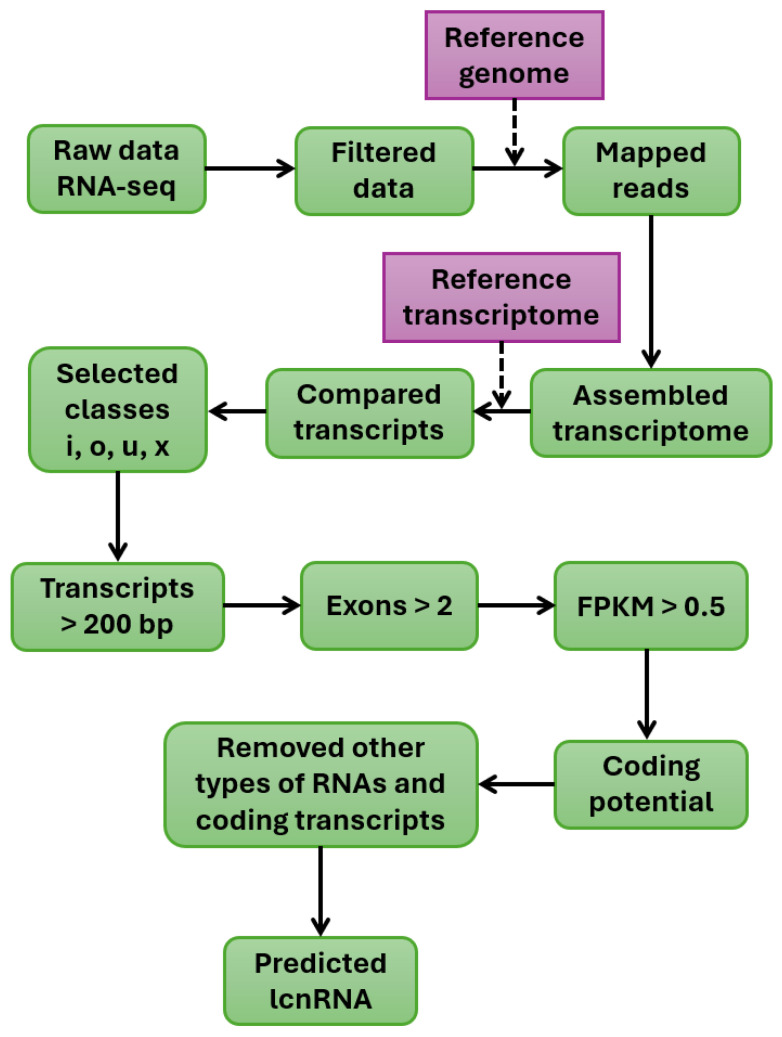
Bioinformatics pipeline of long noncoding RNA (lncRNA) analysis generated from next-generation sequencing (NGS).

A robust discovery workflow begins with quality control of raw reads to remove adapters and low-quality bases (Figure 4). This typically uses Trimmomatic or the all-in-one preprocessor FastP [174,175]. The cleaned reads are then aligned to the reference genome and assembled into transcript models. The widely adopted “new Tuxedo” pipeline—HISAT2 for spliced alignment followed by StringTie for reference-guided assembly—provides sensitive transcript reconstruction and abundance estimates [176]. To annotate novel transcripts and their genomic context, assembled transcripts are compared to the reference annotation via gffcompare (or the legacy program Cuffcompare), which assigns class codes that describe the relationships between the transcripts and their genomic locations. For lncRNA discovery, plant studies commonly retain the intergenic (u), intronic (i), antisense exonic (x), and ambiguous exonic-overlap (o) classes, reflecting novel intergenic, intronic, or antisense candidates (sometimes alongside e, j, and p classes) [177,178,179]. Canonical filters then enforce a minimum length of ≥200 nucleotides (nt), a multiexon structure of ≥2 exons, and an expression threshold. FPKM ≥ 0.5 is a standard threshold used in plant lncRNA catalogs, although cutoff values vary by study and design [180]. Because TPM is sample comparable by design, some pipelines use TPM-based filters or report both metrics [181,182]. The noncoding status of candidates is assessed by coding-potential classifiers, such as CPAT, CPC2, and COME, followed by the exclusion of any transcript bearing known protein domains (Pfam) or matching structured noncoding RNA families (Rfam) [148,149,183].

Additional decontamination steps, via database screens, remove miRNAs, rRNAs, tRNAs, sn/snoRNAs, and pseudogenes [183]. Functional inference integrates multiple orthogonal signals. *cis* hypotheses are prioritized by proximity to neighboring protein-coding genes. *Trans* candidates emerge from coexpression networks and, when available, from predictive RNA–RNA interaction or RNA–binding protein evidence. Network construction and module interrogation are typically performed in Cytoscape, followed by GO/KEGG enrichment to contextualize putative target sets [184]. Plant-specific repositories then facilitate cross-study synthesis—PLncDB v2.0 aggregates 13,834 RNA-seq libraries across 80 species with expression and coexpression tracks. CANTATAdb 3.0 expands curated plant lncRNAs across 108 species. RNAcentral provides a unifying, cross-resource index of noncoding RNA (ncRNA) sequences and structures [45,150]. The best practice is to pair the aforementioned discovery pipeline with orthogonal validation, such as RT–qPCR for expression, smFISH for subcellular localization, and perturbation genetics (CRISPRi/a) for mechanism, while transparently reporting parameter choices, such as class codes, exon/length and expression thresholds, and coding-potential cut-offs, to ensure reproducibility across species and studies.

## 9. Prospects

Research on long noncoding RNAs in plants is rapidly progressing. It is moving from descriptive cataloging to analysis of their role in development. Moreover, studies of the role of these genes in stress adaptation are needed. Moreover, an analysis of their role in intercellular communication is needed. Future research will increasingly integrate multiomic approaches, combining transcriptomics, epigenomics, proteomics, and metabolomics to outline the full spectrum of lncRNA-dependent regulatory networks. High-resolution spatial transcriptomics, single-cell RNA sequencing, and advanced live-cell imaging will be essential for mapping the dynamic localization and movement of lncRNAs in tissues and developmental stages. The discovery of the structural basis of interactions between lncRNAs and proteins, DNA, and other RNAs is important. The secondary and tertiary structures underlying functional specificity can be elucidated via cryo-electron microscopy and computer modeling on the basis of chemical probing. This structural knowledge will enable the rational design of synthetic lncRNA imitators or inhibitors for crop improvement. Another emerging field is the interspecific mobility of lncRNAs, especially in plant–microbe and plant–insect interactions. Understanding how lncRNAs are selectively packaged in extracellular vesicles or bound by RNA-binding proteins for long-distance transport could provide insight into plant defense and communication strategies. This knowledge could contribute to the development of RNA-based biocontrols or targeted delivery systems. The role of lncRNAs in classical signaling and transcriptional networks remains underexplored. Genome editing tools such as CRISPR/Cas13 and CRISPR-mediated epigenome editing enable the targeted perturbation of lncRNA loci and chromatin states. This facilitates causal inference in functional studies. When combined with AI-based predictive modeling, these approaches can reveal inaccessible regulatory nodes inaccessible through conventional gene-by-gene analyses. From a practical standpoint, identifying stress-sensitive lncRNAs provides an opportunity to develop molecular markers for breeding climate-resilient crops. Additionally, engineering lncRNAs that fine-tune hormone signaling, nutrient uptake, or pathogen resistance could provide sustainable agricultural solutions under changing environmental conditions. However, realizing these prospects will require addressing challenges such as low levels of interspecies sequence conservation, complex expression patterns, and the need for functional validation in native contexts. Collaborations that combine molecular biology, structural biology, computational modeling, and field agronomy are critical to translating fundamental discoveries into practical applications.

This structural knowledge will enable the rational design of synthetic long noncoding RNA mimics or inhibitors for crop improvement. Another area of development is the mobility of plant lncRNAs, particularly in the context of plant–microbe and plant–insect interactions. Understanding how lncRNAs are selectively packaged in extracellular vesicles or bound by RNA-binding proteins for long-distance transport may provide insight into plant defense and communication strategies.

## Figures and Tables

**Table 1:** Summarizing long non-coding RNAs (lncRNAs) and their function from various plant species.

lncRNA Name	Plant Species	Biological Function	References
MISSEN (XLOC_057324)	*Oryza sativa*	Imprinted lncRNA in the endosperm; acts as a “decoy” for HeFP helicase protein, disrupts microtubule polymerization, regulates nuclear division rate and grain size	[85]
asDOG1	*Arabidopsis thaliana*	Antisense transcript of DOG1; recruits PRC2 and silences DOG1, regulating seed dormancy depth	[86,87]
MUSHER	*Arabidopsis thaliana*	Integrates ABA and DOG1 signaling; strengthens seed dormancy under stress conditions (drought, high temperature)	[88]
AtR8	*Arabidopsis thaliana*	Pol III-dependent lncRNA; interacts with WRKY46, activates AtEM6 gene, increases ABA sensitivity and enhances dormancy	[89]
TraesLNC1D26001.1	*Triticum aestivum*	Activates TaABI5; delays germination by reinforcing ABA signaling; potential ceRNA role	[90]
VIVIPARY	*Oryza sativa*	Forms a complex with OsMSI1 and OsHDAC1; suppresses ABA signaling, promotes germination and pre-harvest sprouting	[91]
HID1 (HIDDEN TREASURE 1)	*Arabidopsis thaliana*	Red-light induced; binds to NCED9 intron, suppresses ABA biosynthesis and promotes GA biosynthesis, facilitating germination	[2,92,93]
SVR (Seed Vigor-Related)	*Oryza sativa*	Endosperm-specific lncRNA; regulates seed vigor via neighboring SAUR genes involved in auxin metabolism	[94]
WSGAR	*Triticum aestivum*	lncRNA cleaved by miR9678; regulates ABA/GA balance, influences resistance to pre-harvest sprouting	[87]
lnc663	*Triticum aestivum*	Acts as ceRNA for miR1128; derepresses PDAT-like gene, modulates embryo lipid metabolism	[95]
COOLAIR	*Arabidopsis thaliana*	Regulates the flowering locus C (FLC) gene during vernalization. It plays a key role in controlling flowering time	[36,96]
lncRNA_00185	*Metasequoia glyptostroboides*	miR167 decoy, stabilizes RCD1; enhances oxidative stress tolerance and seed viability	[97,98]
LNC_037529	*Oryza sativa*	Produces alternative splicing isoforms associated with seed aging resistance; interacts with repair proteins (e.g., OsXRCC1)	[99]

## Data Availability

No new data were created or analyzed in this study.
